# Comprehensive analysis of anosmin-1 as a potential biomarker and its correlation with epithelial–mesenchymal transition in advanced gastric cancer

**DOI:** 10.1007/s13205-025-04361-y

**Published:** 2025-06-21

**Authors:** Xuan Zhou, Yue Pan, Hong Li, Yi-Fei Sun, Yu-Jun Li, Yan-Xia Jiang, Ting Liu

**Affiliations:** 1 Department of Pathology, the Affiliated Hospital of Qingdao University, 16 Jiangsu Road, Qingdao, 266000 People’s Republic of China; 2 Department of Pathology, Beijing Ditan Hospital, Capital Medical University, No. 8 Jing Shun East Street, Chaoyang District, Beijing, 100015 China

**Keywords:** ANOS1, Prognosis, Immunity, Epithelial–mesenchymal transition, Gastric cancer

## Abstract

Anosmin-1 (ANOS1) is an extracellular matrix (ECM)-related glycoprotein that is highly expressed in a variety of tumors. However, the specific role and mechanism of ANOS1 in advanced gastric cancer (GC) and its correlation with epithelial–mesenchymal transition (EMT) are not well understood, despite ongoing research into the expression of ANOS1 and its impact on clinical outcomes. The ANOS1 expression and clinical data were acquired from The Cancer Genome Atlas (TCGA) database to analyze the differential expression of ANOS1 and its prognostic impact in advanced gastric cancer. The expression of ANOS1 and E-cadherin was detected using immunohistochemical (IHC) techniques in 99 cases of advanced gastric cancer (GC) tissues and their adjacent normal tissues. Subsequently, the correlation of ANOS1 with clinicopathological characteristics was performed to discuss using chi-square test. The prognostic value of ANOS1 expression was assessed using Kaplan–Meier survival analysis and Cox regression. The Spearman’s study was conducted to explore the relationship between ANOS1 expression, immunity, and EMT-related genes. Gene set enrichment analysis (GSEA) was utilized to determine the functional enrichment of ANOS1 in gastric cancer and the “pRRophetic”R package was utilized to determine the relationship between ANOS1 expression and drug sensitivity. TCGA data analysis has shown that ANOS1 was significantly overexpressed in advanced GC and high ANOS1 expression had a poor prognosis. The IHC analysis showed elevated ANOS1 levels in advanced GC tissues compared to the adjacent tissues. High ANOS1 expression was correlated with tumor infiltration, lymph node metastasis, TNM stage, and vascular invasion in advanced GC, indicating its role in tumor invasiveness and metastasis. Furthermore, research indicated that the expression of ANOS1 was inversely associated with E-cadherin levels in advanced GC. Kaplan–Meier analysis demonstrated that patients with high ANOS1 expression had poorer overall survival than those with low expression. Furthermore, ANOS1 was positively associated with EMT-related genes and correlated with 22 types of immune cell infiltration and 7 immune checkpoints. The patients with high expression levels of ANOS1 demonstrated sensitivity to 5-fluorouracil, dasatinib, and docetaxel. GSEA analysis revealed significant enrichment of ANOS1 in multiple oncogenic pathways, particularly extracellular matrix (ECM) receptor interactions, focal adhesion, hematopoietic cell lineage, chemokine signaling pathways, pathways in cancer, and transforming growth factor-β signaling pathway. ANOS1 may impact gastric cancer progression by regulating EMT, suggesting its dual utility as both a prognostic biomarker and a novel therapeutic target for GC intervention.

## Introduction

Cancer is a complex disease characterized by the uncontrolled proliferation and abnormal growth of cells (Mishra et al. 2023a, 2023b). Despite decades of extensive research, cancer continues to pose a major global health challenge (Prajapati et al. 2025). Gastric cancer (GC) is the fifth most prevalent malignant tumor worldwide, with an annual incidence exceeding 1 million cases and accounting for approximately 769,000 deaths annually. The male-to-female incidence ratio is approximately 2:1 (Sung et al. 2021; Smyth et al. 2020). In China, GC is the second leading cause of cancer-related mortality (Chen et al. 2016). Despite the advancements in targeted therapeutic drugs, their impact on patient prognosis remains limited (Soularue et al. 2015; Hsu et al. 2020). The significance of elucidating the underlying mechanisms of GC pathogenesis and discovering innovative molecular therapeutic targets for its treatment is underscored by the increasing incidence and the need for improved therapeutic strategies.

Anosmin-1 (ANOS1) is an extracellular matrix (ECM)-related secreted glycoprotein encoded by the KAL1 gene, which promotes the migration of gonadotropin-releasing hormone neurons from the olfactory substrate to the hypothalamus during early brain development. ANOS1 affects the development of primary and secondary olfactory processing regions (González-Martínez et al. 2004). Previous studies have demonstrated a strong association between ANOS1 and various malignant tumors, such as colon cancer, lung cancer, and ovarian cancer and its abnormal expression promotes tumor cell growth, metastasis, and poor prognosis (Jian et al. 2009; Kim et al. 2014; Qi et al. 2017). Mitsuro Kanda et al. reported that ANOS1 was upregulated in gastric cancer and confirmed ANOS1 as a cell adhesion protein to mediate the EMT through coordinate expression with other EMT-related molecules in GC cells (Kanda et al. 2016). Epithelial–mesenchymal transition (EMT) is a fundamental biological process involved in embryonic development, wound healing, and fibrotic diseases (Lee et al. 2006). Increasing evidence demonstrated the critical role of abnormal EMT activation in tumor occurrence, invasion, and metastasis, including GC (Zhao et al. 2013; Montemayor-Garcia et al. 2013; Ren et al. 2018). This process is characterized by the loss of cell polarity and adhesion molecule function (such as E-cadherin), and enhanced function of mesenchymal proteins (such as vimentin) (Suzuki et al. 2017). So we studied whether ANOS1 participated EMT process in GC.

The tumor microenvironment (TME) comprises various elements such as immune cells, inflammatory mediators, endothelial cells, mesenchymal cells, and ECM molecules (Hanahan and Coussens 2012). The immune cell infiltration in the TME directly impacts tumor development and progression. TME serves as the critical factor for tumors, encompassing invasive immune cells, and stromal components. Accumulating evidence demonstrated that immune cell infiltration plays a pivotal role in both tumor progression and the efficacy of immunotherapy (Rad et al. 2021). Consequently, investigating the relationship between ANOS1 and tumor-associated immune cell infiltration is critically important. Our study aimed to investigate the expression, clinical significance, prognosis, immunity, and drug sensitivity of ANOS1 in advanced GC. Additionally, we aimed to explore the interaction between ANOS1 and the EMT process, providing a new theoretical basis for clarifying the role of ANOS1 in GC progression.

## Materials and methods

### Data acquisition

The expression profile and clinical data of ANOS1 in advanced GC were obtained from The Cancer Genome Atlas database (TCGA database, STAD dataset). The patients with stage II–IV was included in the study and the patients with stage I was not included in the work. The Wilcoxon test was employed to assess the differential expression of ANOS1 between advanced GC tissues and normal tissues. Kaplan–Meier analysis was performed to evaluate the effect of ANOS1 expression on prognosis in advanced GC.

### Collection samples

Paraffin-embedded advanced GC specimens were collected from 99 patients admitted at the Affiliated Hospital of Qingdao University between 2014 and 2015. The study was authorized by local institutional review boards (batch number: No. ZR2022MH148) and written informed consent was obtained from all patients before surgery. The specimens included advanced GC tissues and their adjacent tissues. The patients had no history of preoperative radiotherapy or chemotherapy and had complete clinical and pathological data. The cohort consisted of 65 men and 34 women, with an age range of 23–85 years. The cohort comprised 99 gastric cancer patients with the following histopathological subtypes: moderately differentiated (*n* = 34), poorly differentiated (*n* = 65). According to histological morphology, 99 patients were divided into two groups: the high-adhesion group (including 30 patients with tubular adenocarcinoma, 10 with papillary adenocarcinoma, and 27 with mucinous adenocarcinoma) and the low-adhesion group (including 18 with signet-ring cell carcinoma and 14 with poorly cohesive carcinoma). Moreover, the lymph node metastasis tissues were randomly selected from 40 patients with lymph node metastasis among the 99 individuals with advanced GC.

### Immunohistochemical staining

The specimens were embedded and cut into 4-μm sections, followed by antigen retrieval solution repair (pH = 8.0). The endogenous peroxidase activity was inactivated using a 3% H_2_O_2_ solution. Subsequently, ANOS1 antibody (1:200 dilution, Abcam, UK) and E-cadherin antibody (1:100, Fuzhou Maixin Biotechnology Co., Ltd.) were added and incubated overnight at 4 ℃. Then, the secondary antibody (PV-6000, Beijing Zhongshan Jinqiao Biological Co., Ltd.) was added, followed by DAB staining and hematoxylin counterstaining. ANOS1 was localized in the cytoplasm and 500 cells were counted in each high-power field. Pathological scoring was conducted by two experienced pathologists. The ANOS1 staining results were interpreted based on the percentage of stained cells and staining intensity. The staining percentages were categorized as no staining (0%–5%, 0 point), minimal staining (6%–30%, 1 point), focal staining (31%–70%, 2 points), and diffuse staining (> 70%, 3 points). The staining intensity was categorized as no staining (0 point), light yellow (1 point), brown yellow (2 points), and brown (3 points). The final score was determined by multiplying these scores, with a score of ≤ 2 indicating low expression and a score of > 2 indicating high expression (Kanda et al. 2014). The evaluation criteria for E-cadherin were based on two main components: the percentage of stained cells and staining intensity. The staining percentages were categorized as no staining (0%–5%, 0 point), minimal staining (6%–25%, 1 point), focal staining (26%–50%, 2 points), and diffuse staining (> 50%, 3 points). The staining intensity was categorized as no staining (0 point), light yellow (1 point), brown yellow (2 points), and brown (3 points). The final score was determined by multiplying these scores. A score of ≤ 3 points was classified as low expression, while a score of > 3 points was classified as high expression (Zhang et al. 2004).

### Function enrichment of ANOS1 in GC

Differentially expressed genes were identified using DESeq2 in R, according to the median expression of ANOS1. Gene set enrichment analysis (GSEA) was employed to investigate the molecular function of ANOS1. We selected KEGG for studying the molecular function of ANOS1 and we displayed six significantly enriched pathways in descending order of normalized enrichment scores (NES) values. FDR < 0.25 indicates that the enrichment analysis is statistically significant.

### Relationship between ANOS1 and EMT in advanced GC

The EMT process significantly contributes to the disruption of cellular adhesion in cancer cells, thereby facilitating tumor invasion, metastasis, and other related phenomena. The Spearman’s correlation analysis was performed using data from the TCGA database to explore the relationship between ANOS1 with EMT-related genes (CDH2, VIM, SNAIL1, TWIST1, ZEB1, and ZEB2) at mRNA level in GC.

### Correlation of ANOS1 with immunity in GC

The single-sample GSEA (ssGSEA) algorithm was employed to examine the association between ANOS1 expression and 24 distinct types of immune cells infiltration. The ESTIMATE tool was used to analyze the relationship between gene expression and stromal score, immune score, and ESTIMATE score. Additionally, Spearman’s analysis was conducted to investigate the correlation between ANOS1 correlation and common immune checkpoints.

### Drug sensitivity

Eight commonly used chemotherapy drugs were selected to assess the association of ANOS1 expression with drug sensitivity using the “pRRophetic” package. The half-maximal inhibitory concentration (IC50) values of these drugs were utilized to evaluate the drug sensitivity.

### Statistical analysis

All the statistical methods were carried out in the R language (Version:4.2.1). The Wilcoxon rank-sum test was used to analyze the differences in ANOS1 expression between advanced GC and corresponding adjacent tissues, and between primary lesions and lymph node metastases. The Kruskal–Wallis H-rank sum test was used to compare the ANOS1 expression in different pathological gastric tissues and advanced GC tissues with varying degrees of differentiation. The diagnostic ROC was analyzed using the pROC package for the data and the results were visualized using ggplot2 in R language. Additionally, the relationship between ANOS1 expression and clinicopathological features in advanced GC was assessed using the chi-square test. Survival analysis and Cox regression was conducted using “survival” package. The correlation between ANOS1 and E-Cadherin expression in advanced GC was analyzed using the Spearman’s correlation. A *P* value of < 0.05 was considered significant for all tests.

## Results

### Expression, clinical analysis, and prognosis of ANOS-1 in the TCGA database

**Fig. 1:**
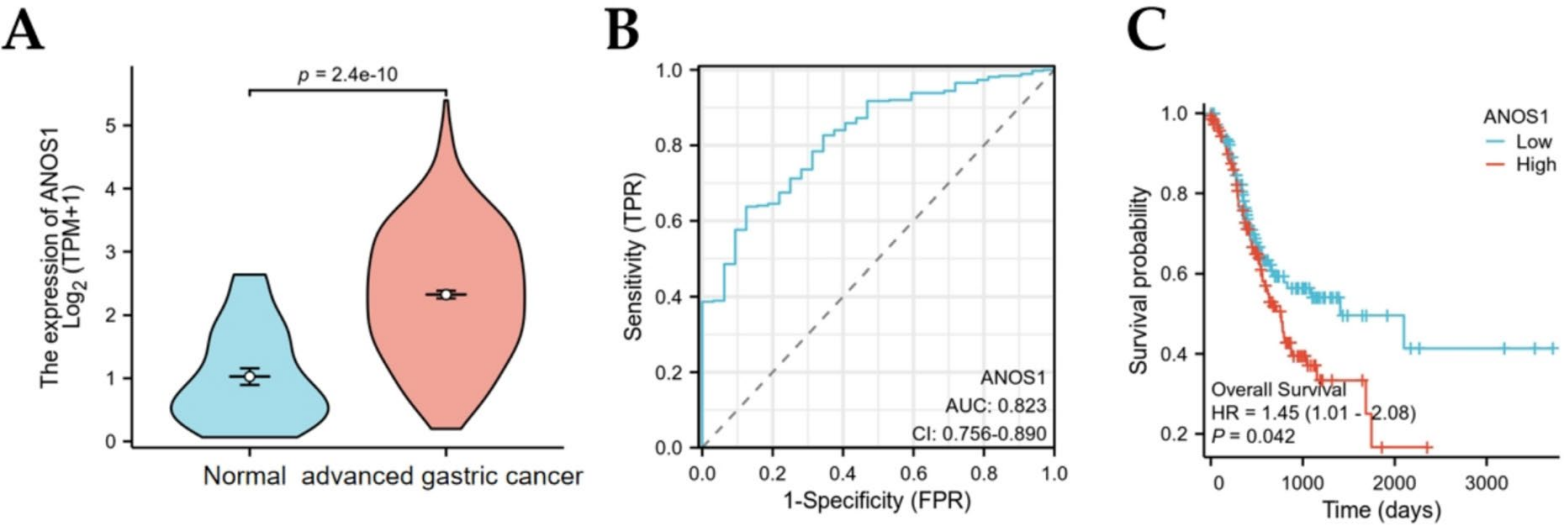
The expression, diagnostic value, and prognosis of ANOS1 expression in the TCGA database. (**A**) ANOS1 was higher expression in advanced gastric cancer than normal tissues. (**B**) The diagnostic ROC of ANOS1 for predicting gastric cancer.(**C**) The high expression of ANOS1 had a poor survival in advanced GC

Utilizing the data from TCGA database, the study revealed significantly elevated ANOS1 expression levels in advanced GC tissues compared with normal tissues (Fig. 1A). The receiver operating characteristic (ROC) value of ANOS1 expression in GC and normal tissues was 0.823, suggesting a significant diagnostic value of ANOS1 in GC (Fig. 1B). Kaplan–Meier curves were employed to assess the impact of ANOS1 expression on overall survival (OS). The analysis showed that patients with high ANOS1 expression exhibited a poorer prognosis compared with those with low ANOS1 expression (Fig. 1C).

### Immunohistochemical analysis of ANOS1 expression in advanced GC and its association with clinicopathological characteristics

**Fig. 2:**
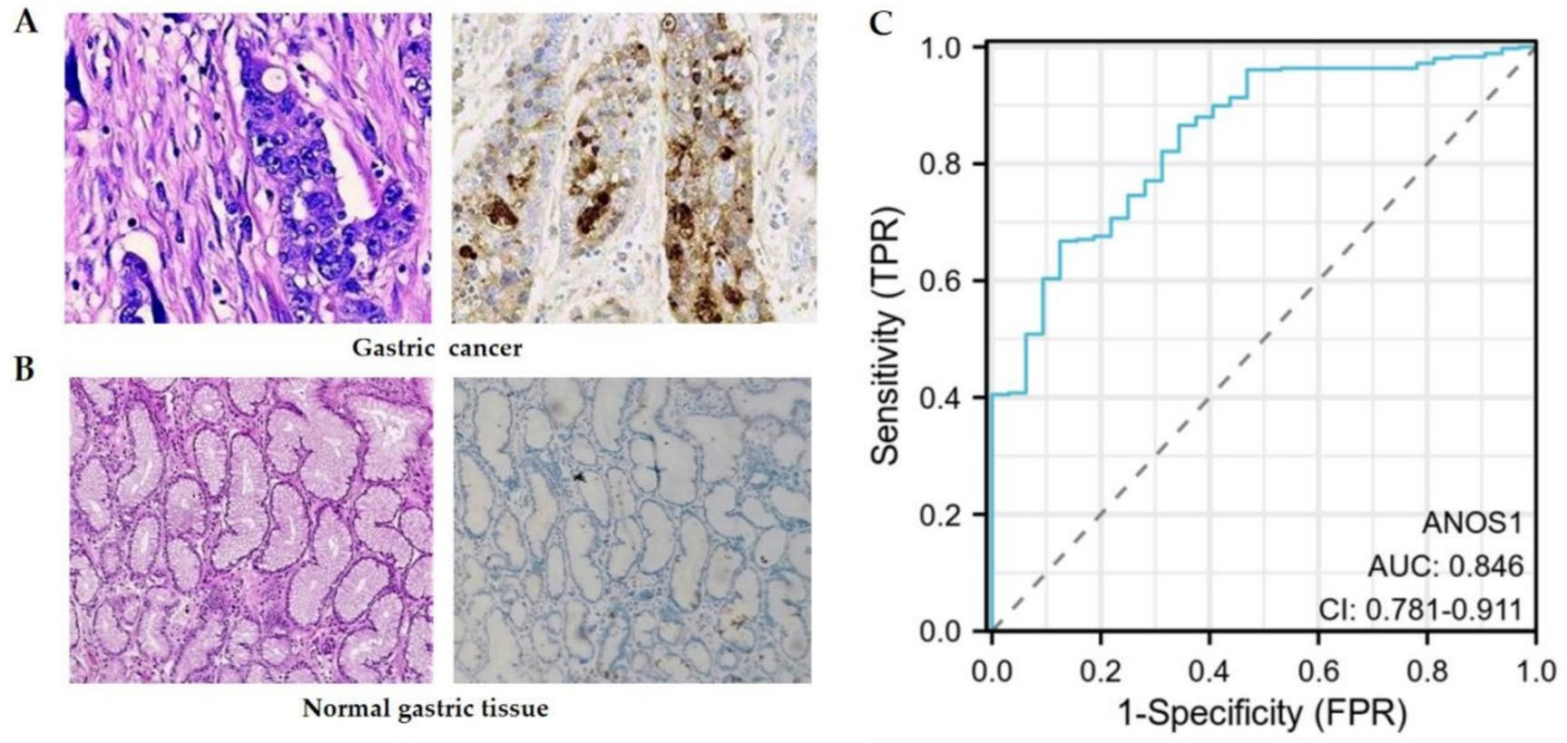
The differential expression and diagnostic ROC of ANOS1 in advanced GC and normal tissues. (**A**, **B**) ANOS1 expression was higher in advanced GC than normal tissues. (**C**) The AUC was 0.846, indicating higher diagnostic value in advanced GC

The ANOS1 expression was analyzed in advanced GC and adjacent tissue samples from 99 patients. The findings revealed a markedly elevated level of ANOS1 expression in advanced gastric cancer tissues relative to adjacent normal tissues (*P* < 0.001) (Fig. 2A and B, Table 1). The diagnostic ROC curve showed that the AUC was 0.846, indicating that it has good diagnostic value in distinguishing gastric cancer from normal tissues (Fig. 2C). Furthermore, the relationship between ANOS1 expression and clinicopathological features was further investigated. The ANOS1 expression was significantly higher in patients with T3–T4, positive lymph node metastasis, positive vascular invasion, and stage III–IV than those with T2, negative lymph node metastasis, negative vascular invasion, and stage II (85.71% vs. 20.00%; 83.95% vs. 38.89%; 96.88% vs. 65.67%; 82.86% vs. 58.62%, respectively; *P* < 0.001). However, no significant correlation was observed between ANOS1 expression and the patient’s sex, age, tumor differentiation, and tumor size (*P* > 0.05) (Table 2).

**Table 1:** ANOS-1 expression in advanced gastric cancer and corresponding adjacent tissues

	N	ANOS1		*Z*	*P*
		High expression	Low expression		
Cancer tissue	99	75	24	−8.485	<0.001
Normal tissue	99	3	96

**Table 2:** The relationship of ANOS1 expression with clinical pathological features in advanced gastric cancer

Variable	*n*	ANOS1 expression		*χ*^2^	*P*
		Low (%)	High (%)		
Gender				3.444	0.084
Male	65	12 (18.46)	53 (81.54)		
Female	34	12 (35.30)	22 (64.70)		
Age				0.454	0.500
≥ 60	43	9 (20.93) 9	34 (79.07)		
<60	56	15 (26.79)	41 (73.21)		
Differentiation degree				0.753	0.385
Middle differentiation	34	10 (29.41)	24 (70.59)		
Low differentiation	65	14 (21.54)	51 (78.46)		
Tumor size				0.099	0.753
≥ 5 cm	55	14 (25.45)	41 (74.55)		
<5 cm	44	10 (22.73)	34 (77.27)		
Tumor infiltration				26.455	<0.001
T2	15	12 (80.00)	3 (20.00)		
T3 ~ T4	84	12 (14.29)	72 (85.71)		
TNM stage				6.558	0.010
II	29	12 (41.37)	17 (58.62)		
III-IV	70	12 (17.14)	58 (82.86)		
Lymph node metastasis				16.283	<0.001
+	81	13 (16.05)	68 (83.95)		
−	18	11 (61.11)	7 (38.89)		
Vascular invasion				11.481	<0.001
+	32	1 (3.12)	31 (96.88)		
−	67	23 (34.33)	44 (65.67)		

### ANOS1 expression in the primary tumors and the corresponding lymph node metastases of advanced GC

**Fig. 3:**
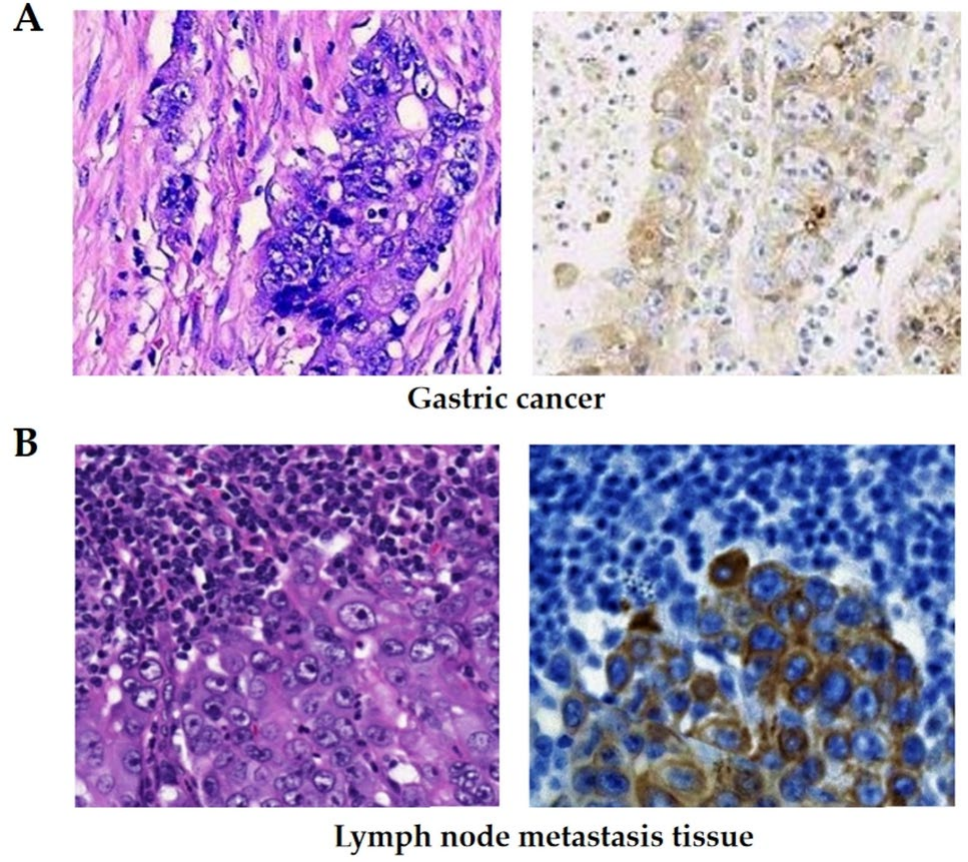
The expression of ANOS1 in the cancer tissues of lymph node metastasis was higher than that in the primary cancer tissues

The association between ANOS1 expression and the clinicopathological characteristics suggested a potential link between ANOS1 and the invasive and metastatic potential of advanced GC. Therefore, we randomly selected 40 patients with the corresponding lymph node metastases from advanced GC and examined the differential expression of ANOS1 in the primary tumor and corresponding metastatic lesions. The analysis revealed significantly higher expression levels of the ANOS1 gene in lymph node metastases compared to primary tumors. (Fig. 3A and B, Table 3).

**Table 3:** The expression of ANOS1 in primary and lymph node metastases of gastric cancer

	*n*	ANOS1		*Z*	*P*
		High expression	Low expression		
Primary lesion	40	24	16	−5.786	<0.001
Metastatic lesion	40	31	9		

### Expression of ANOS1 and E-cadherin in advanced GC of different histological types

**Fig. 4:**
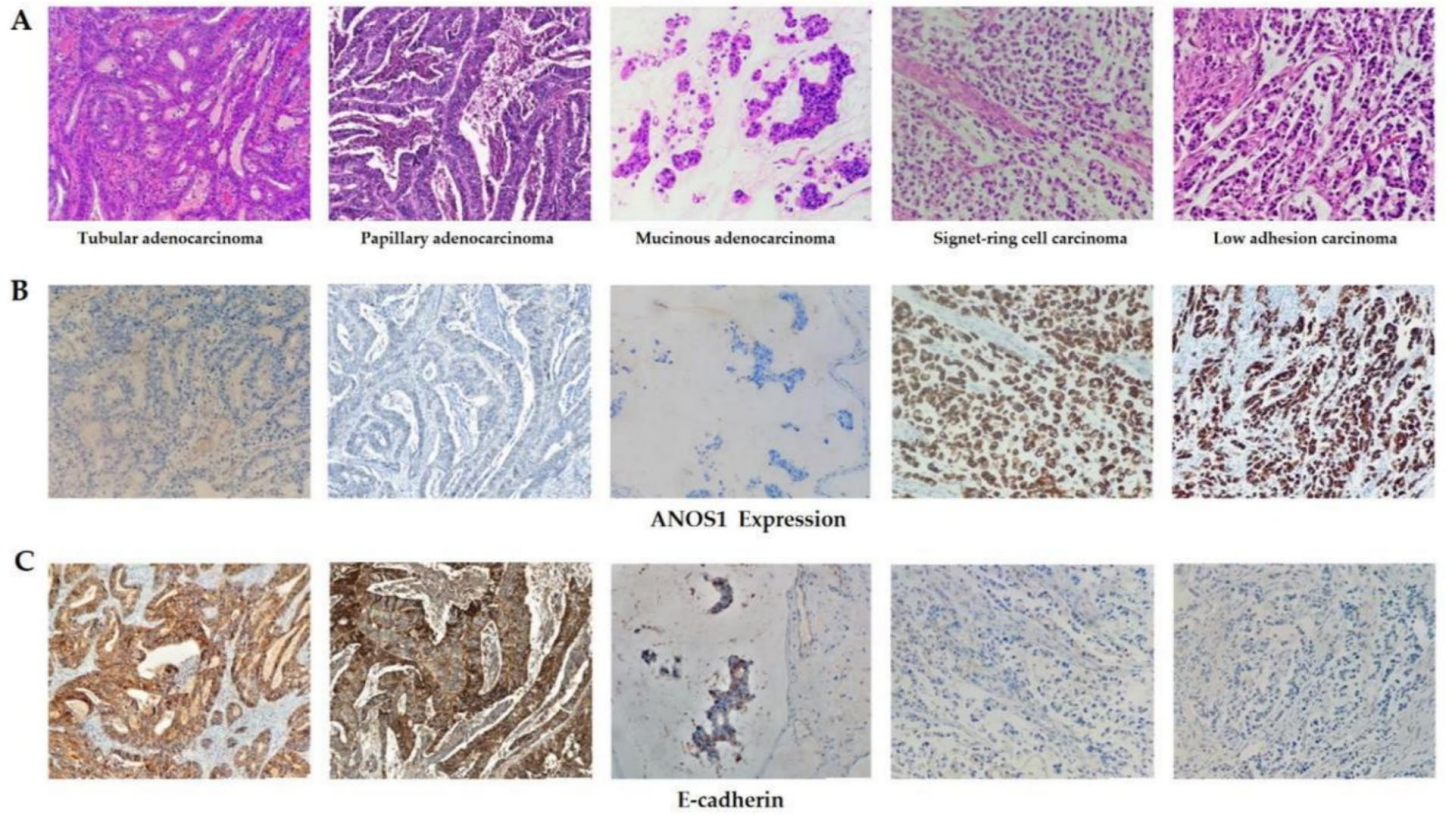
The expression of ANOS1 (**B**) and E-cadherin (**C**) in tubular adenocarcinoma, papillary adenocarcinoma, mucinous adenocarcinoma, signet-ring cell carcinoma and low adhesion carcinoma

A total of 99 patients with advanced GC were divided into two groups based on histological morphology: the high-adhesion group (including 30 patients with tubular adenocarcinoma, 10 with papillary adenocarcinoma, and 27 with mucinous adenocarcinoma) and the low-adhesion group (including 18 with signet-ring cell carcinoma and 14 with poorly cohesive carcinoma). The ANOS1 expression was analyzed in different histological types, revealing a significant difference in ANOS1 expression among them (*P* < 0.001). No significant differences were found in ANOS1 expression among tubular adenocarcinoma, papillary adenocarcinoma, and mucinous adenocarcinoma tissues (*P* > 0.05). Similar results were observed between signet-ring cell carcinoma and poorly cohesive carcinoma (*P* > 0.05). However, the ANOS1 expression in the low-adhesion group was significantly increased compared with that in the high-adhesion group (*P* < 0.05). These findings suggest a potential correlation between elevated ANOS1 expression and diminished tumor cell adhesion (Fig. 4A and B, Table 4). Conversely, the E-cadherin expression demonstrated an inverse trend in advanced GC tissues across different histological types. The E-cadherin expression in the low-adhesion group was significantly lower than that in the high-adhesion group (Fig. 4C, Table 5).

**Table 4:** Expression of ANOS1 in advanced gastric cancer of different differentiation types

Differentiation type	*n* = 99	Scoring ratio of ≥ 4 points	*Z*	*P*
Tubular adenocarcinoma	30	26.7%	29.416	<0.001^a^
Papillary adenocarcinoma	10	30%	−0.135	0.892^b^
Mucinous adenocarcinoma	27	33.3%	−1.163	0.245^c^
			−1.670	0.098^d^
Signet ring cell carcinoma	18	66.7%	−2.352	0.019^e^
Poorly cohesive carcinoma	14	78.6%	−0.717	0.474^f^

^a^Comparison of five groups. *H* = 25.77

^b^Tubular adenocarcinoma and Papillary adenocarcinoma

^c^Papillary adenocarcinoma and Mucinous adenocarcinoma

^d^Tubular adenocarcinoma and Mucinous adenocarcinoma

^e^Mucinous adenocarcinoma and Signet ring cell carcinoma

^f^Signet ring cell carcinoma and poorly cohesive carcinoma

**Table 5:** The E-cadherin expression in advanced gastric cancer with different differentiation types

Differentiation type	*n* = 99	Scoring ratio greater than 3 points	*Z*	*P*
Tubular adenocarcinoma	30	53.3%	21.933	<0.001^a^
Papillary adenocarcinoma	10	40%	−0.061	0.951^b^
Mucinous adenocarcinoma	27	37%	−0.071	0.944^c^
			−0.898	0.369^d^
Signet ring cell carcinoma	18	16.7%	−2.464	0.014^e^
Poorly cohesive carcinoma	14	14.3%	−0.792	0.428^f^

^a^Comparison of five groups. *H* = 21.95

^b^Tubular adenocarcinoma and Papillary adenocarcinoma

^c^Papillary adenocarcinoma and Mucinous adenocarcinoma

^d^Tubular adenocarcinoma and Mucinous adenocarcinoma

^e^Mucinous adenocarcinoma and Signet ring cell carcinoma

^f^Signet ring cell carcinoma and poorly cohesive carcinoma

### Correlation analysis of ANOS1 and E-cadherin expression in advanced GC

**Fig. 5:**
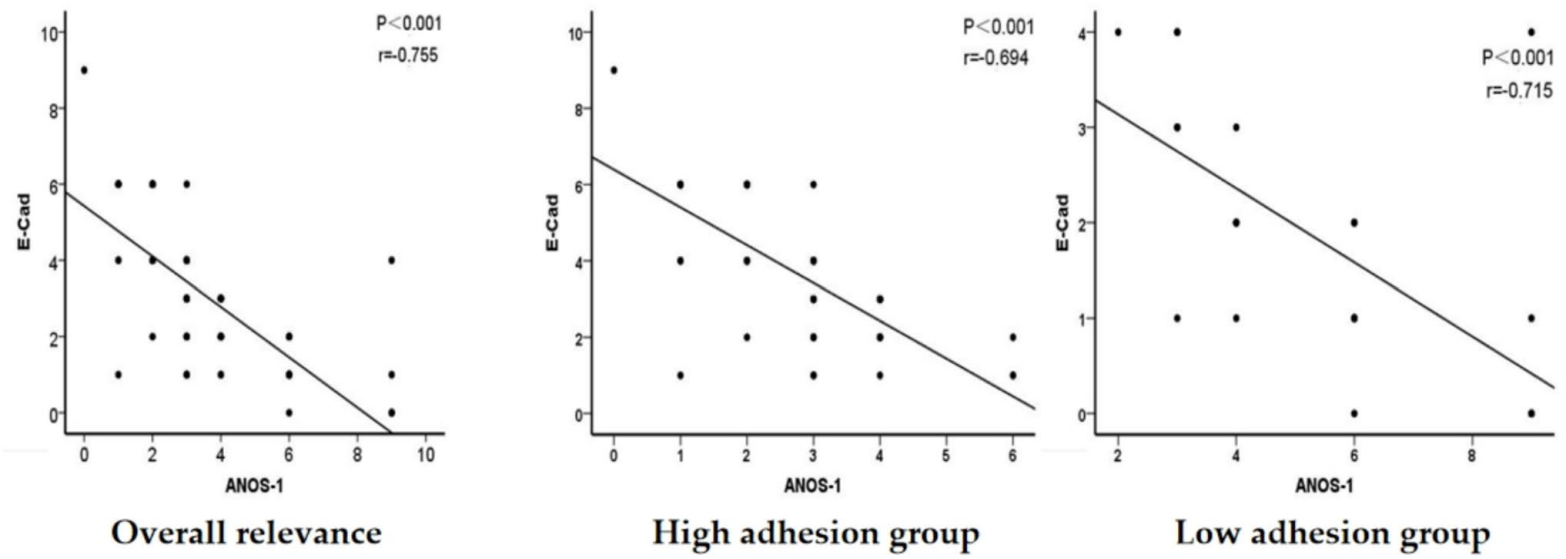
Correlation analysis of ANOS1 with E-cadherin in advanced GC, high adhesion group, and low adhesion group

To further explore the association between reduced tumor cell adhesion and ANOS1 expression, Spearman’s correlation analysis was performed to evaluate the correlation between ANOS1 and E-cadherin expression in advanced GC. Among 99 patients with advanced GC, 53 with high ANOS1 expression exhibited low E-cadherin expression, while 13 with low ANOS1 expression showed high E-cadherin expression. ANOS1 expression was significantly negatively correlated with E-cadherin in advanced GC (*r* = −0.755, *P* < 0.001) (Table 6). Furthermore, linear regression analysis and scatter plots showed a significant negative correlation between ANOS1 and E-cadherin expression in both groups (Fig. 5).

**Table 6:** Spearman correlation analysis of ANOS-1 and E-Cadherin in advanced gastric cancer

ANOS1	E-Cadherin		*R*	*P*
	Low expression	High expression		
Low expression	11	13	−0.755	<0.001
High expression	53	22

### Relationship between ANOS1 expression and prognosis in patients with advanced GC

**Fig. 6:**
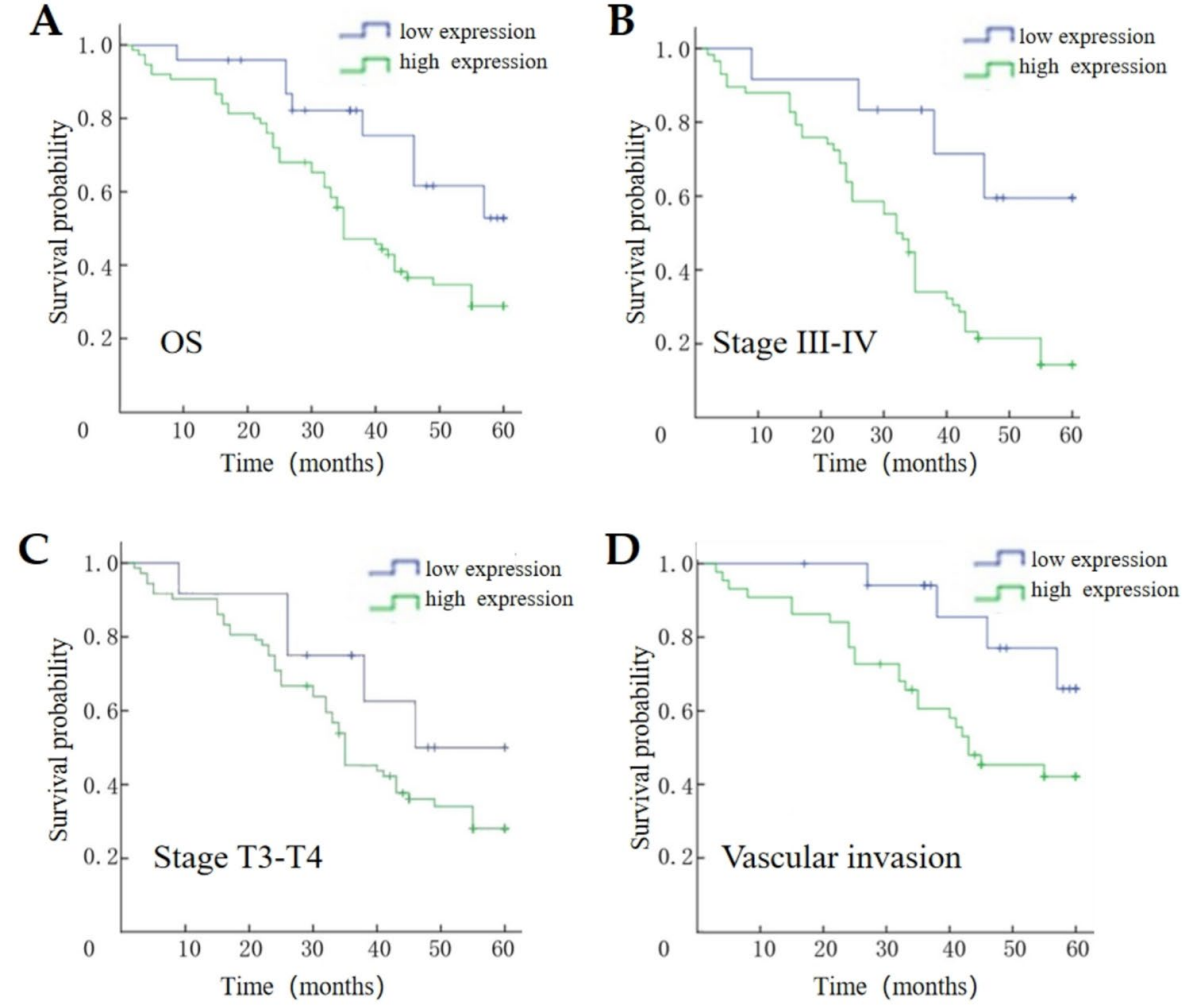
The prognosis of ANOS1 in advanced GC. (**A**) High expression of ANOS1 had a poorer outcome than low expression of ANOS1. (**B**) In stage III-IV group, high expression of ANOS1 had a poor survival. (**C**) In stage T3-T4, high expression of ANOS1 had a unfavorable outcome. (**D**) High expression of ANOS1 had a worse prognosis in patients with vascular invasion

**Fig. 7:**
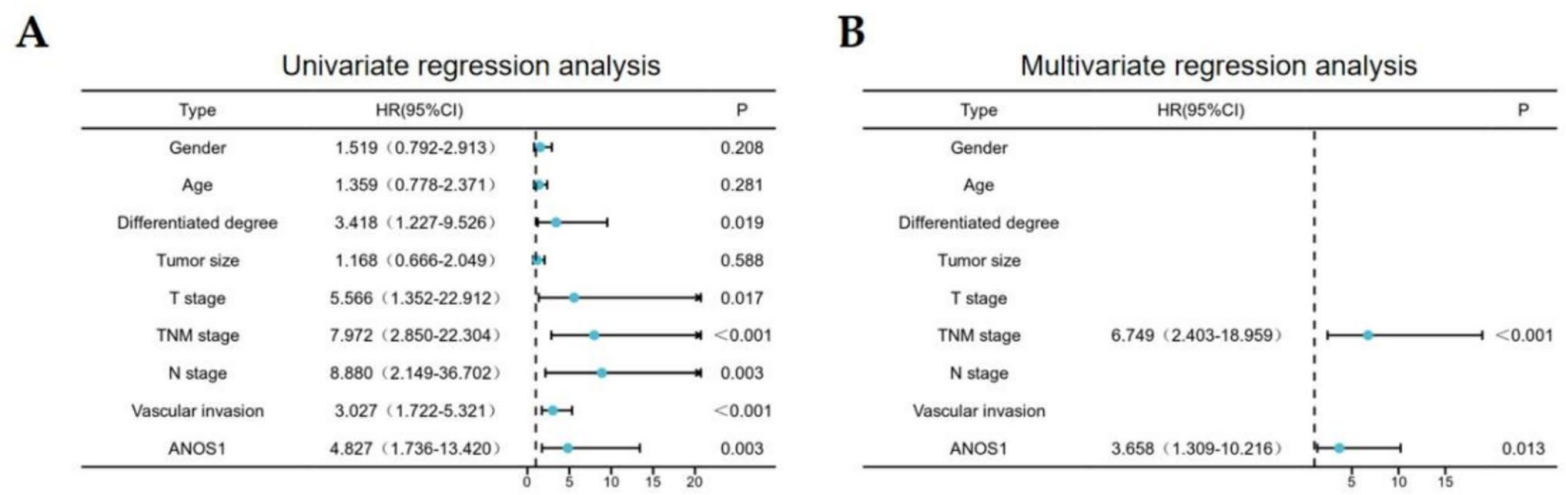
Univariate (**A**) and multifactorial analysis (**B**) of ANOS1 expression in advanced GC and its effect on OS

Patients with elevated ANOS1 overexpression experienced poorer OS compared with those with low expression (Fig. 6A). Specifically, individuals with elevated ANOS1 expression demonstrated poorer OS compared with those with low ANOS1 expression within the stage III–IV, T3–T4, and vascular invasion subgroups (Fig. 6B–D). Furthermore, univariate and multivariate Cox regression analyses were conducted to investigate the impact of ANOS1 expression on prognosis. Univariate regression analysis demonstrated that ANOS1 expression (*P* < 0.05), TNM stage (*P* < 0.001), N stage (*P* < 0.01), T stage (*P* < 0.05), differentiation degree (*P* < 0.05), and vascular invasion (*P* < 0.001) had a significant impact on the OS of patients with GC. The multivariate analysis showed that the ANOS1 expression and TNM staging were prognostic factors affecting the OS of advanced GC (Fig. 7).

### Functional enrichment analysis of ANOS1 in GC

**Fig. 8:**
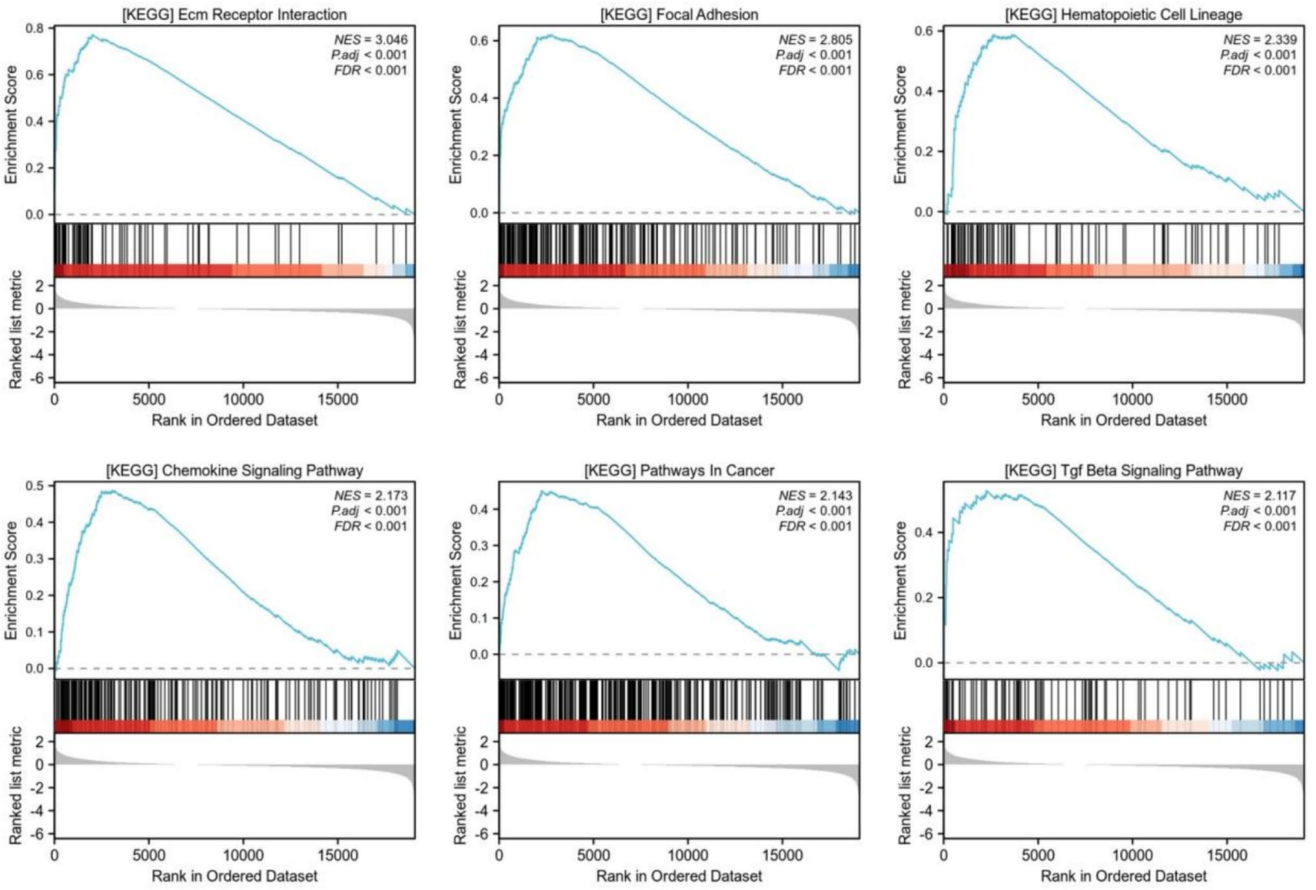
The functional enrichment of ANOS1 in high expression group of GC

To elucidate the biological process associated with ANOS1, GSEA of ANOS1 was conducted in patients with GC. The analysis revealed that ANOS1 is involved in the ECM receptor interaction, focal adhesion, hematological cell lineage, chemokine signaling pathway, pathways in cancer, and transforming growth factor (TGF)-β signaling pathway (Fig. 8).

### Correlation analysis between ANOS1 expression and EMT-related markers CDH2, VIM, SNAIL1, TWIST1, ZEB1 and ZEB2

**Fig. 9:**
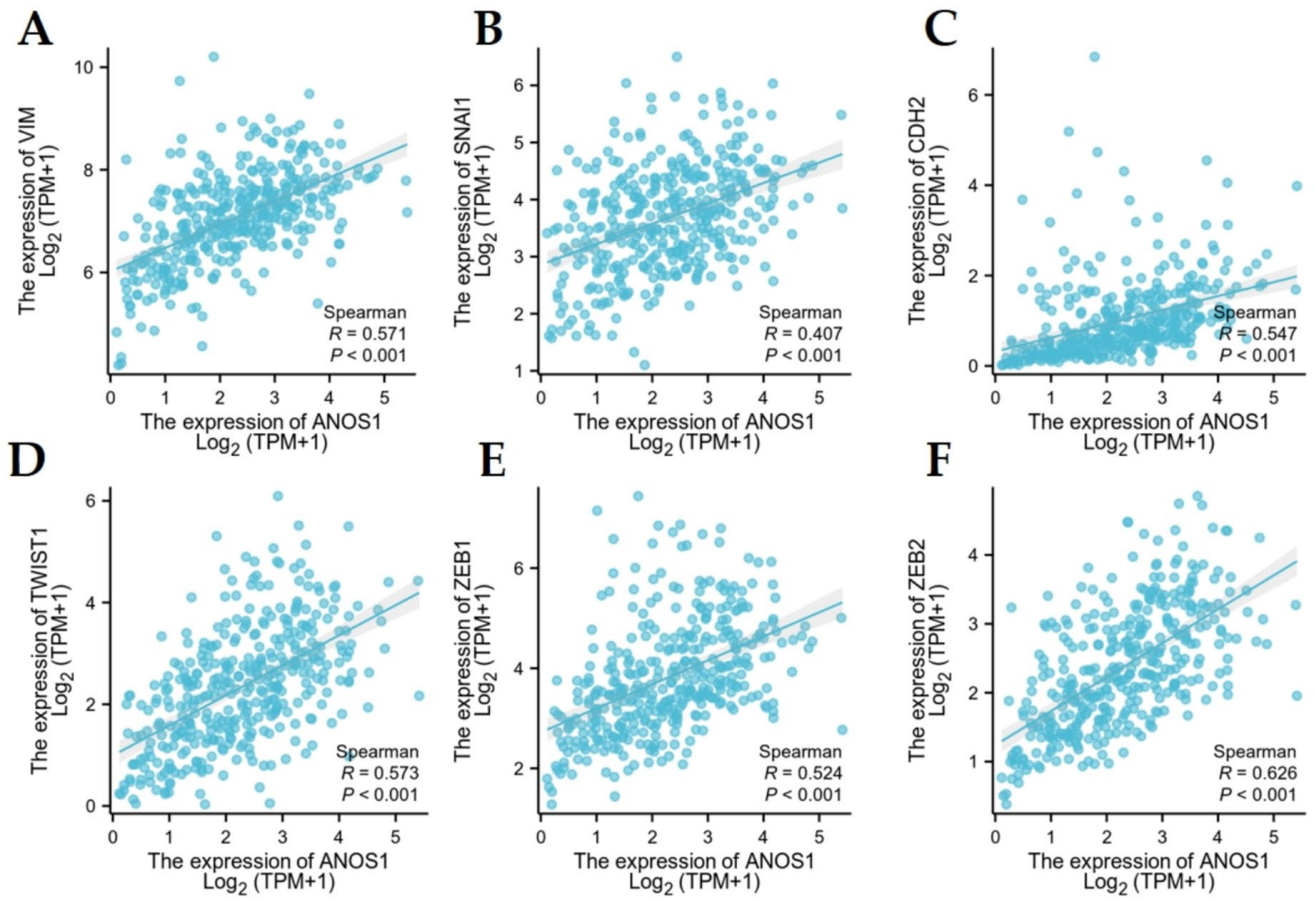
The correlation between ANOS1 and VIM (**A**), SNAI1 (**B**), CDH2 (**C**), TWIST1, (**D**), ZEB1 (**E**), and ZEB2 (**F**) in gastric cancer

The previous findings suggested a significant inverse relationship between ANOS1 and E-cadherin expression in advanced GC, with ANOS1 involvement in the ECM receptor interaction process and focal adhesion. Therefore, we analyzed the association between ANOS1 expression and EMT-related genes (CDH2, VIM, SNAI1, TWIST1, ZEB1, and ZEB2) using the TCGA database. The findings revealed a significant positive association of ANOS1 with CDH2, VIM, SNAI1, TWIST1, ZEB1, and ZEB2 expression (*P* < 0.001), suggesting the potential involvement of ANOS1 in the EMT of GC cells (Fig. 9A–F). These results will serve as a basis for investigating further the molecular mechanisms by which ANOS1 may facilitate tumor invasion and metastasis.

### Correlation between ANOS1 and immunological features in GC

**Fig. 10:**
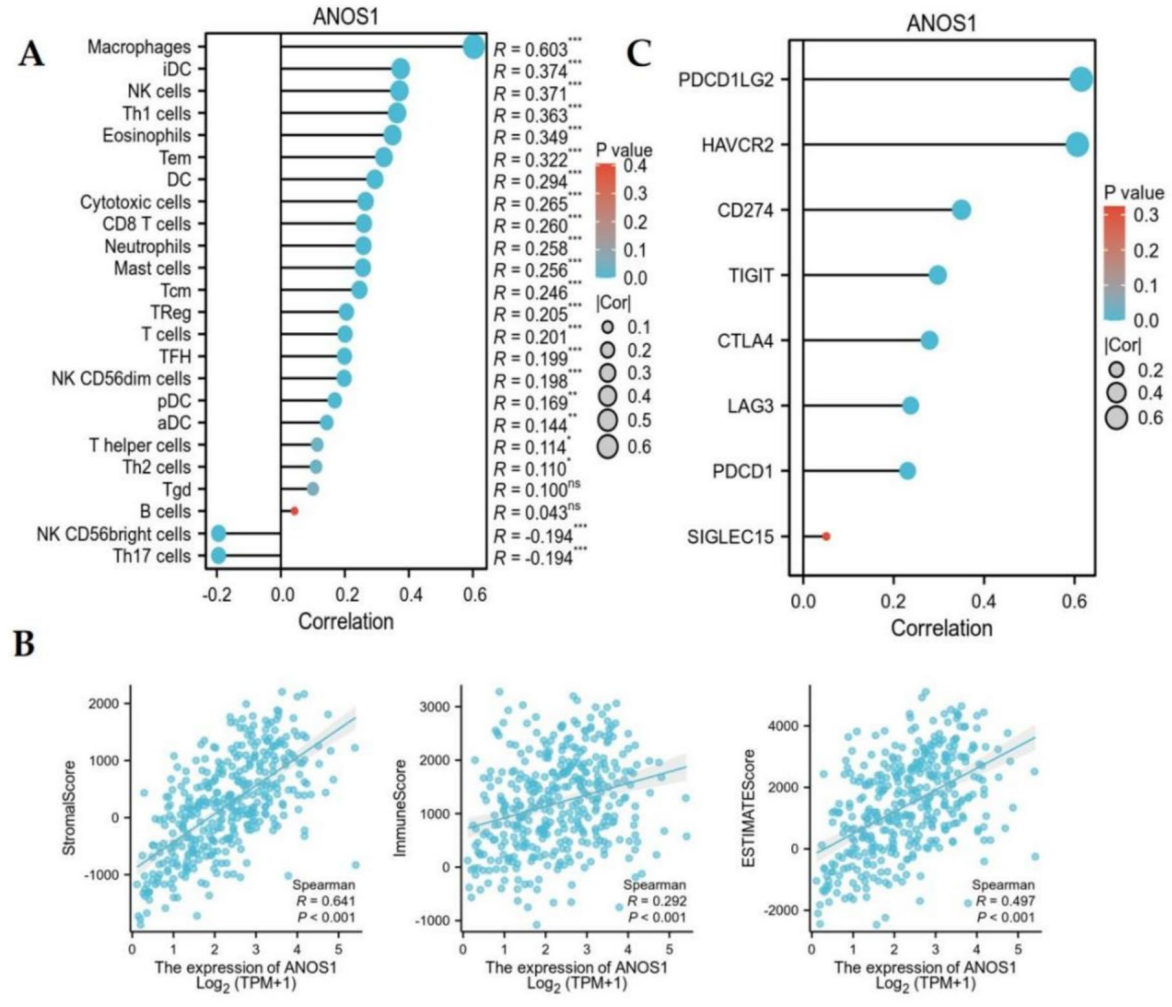
The relationship of ANOS1 with immunity in advanced GC. (**A**) The correlation of ANOS1 with immune cell infiltration using ssGSEA. (**B**) The association of ANOS1 with immunity using ESTIMATE algorithm. (**C**) The correlation of ANOS1 with immune checkpoints

The association between ANOS1 expression and the infiltration of 24 types of immune cells in GC was examined using ssGSEA. The analysis revealed a positive correlation of ANOS1 expression in GC with macrophages, iDC, NK cells, Th1 cells, eosinophils, Tem, DC, cytotoxic cells, CD8 T cells, neutrophils, mast cells, Tcm, TReg, T cells, TFH, NK CD56 dim cells, pDC, aDC, T helper cells, and Th2 cells. By contrast, ANOS1 expression demonstrated a negative correlation with NK CD56 bright cells and Th17 cells (Fig. 10A). The ESTIMATE algorithm was used to evaluate the correlation of stromal score, immune score, and ESTIMATE score with gene expression in tumors. ANOS1 expression in GC was positively correlated with stromal score, immune score, and ESTIMATE score (Fig. 10B). Among them, immune checkpoint inhibitors have shown promising therapeutic effects in various solid tumors, such as non-small cell lung cancer, melanoma, head and neck cancer, and liver cancer. Our results indicated that ANOS1 expression was positively correlated with PDCD1LG2, HAVCR2, CD274, TIGIT, CTLA4, LAG3, and PDCD1 in GC (Fig. 10C).

### Relationship between ANOS1 expression and drug sensitivity in GC

**Fig. 11:**
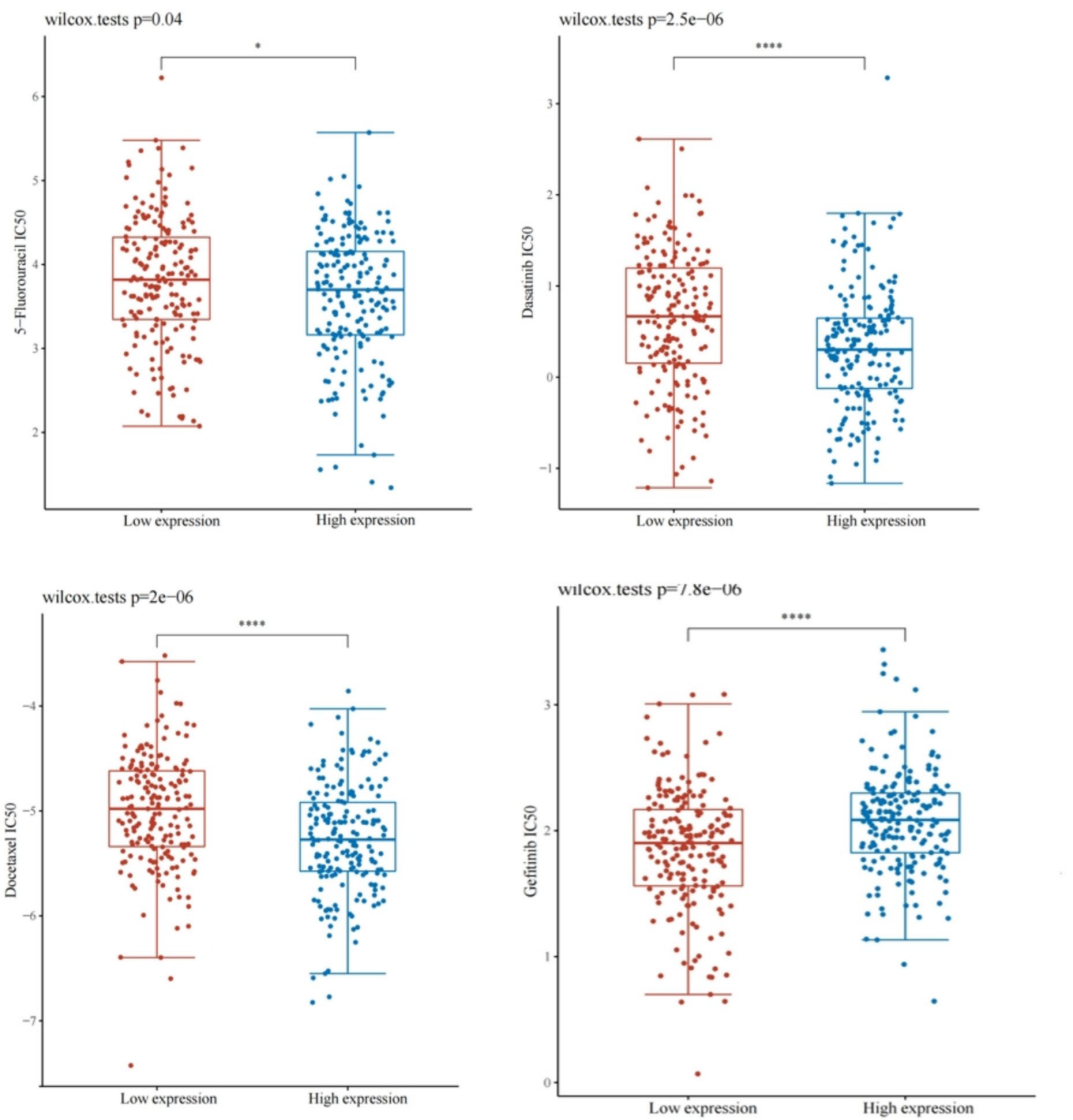
The correlation between ANOS1 and drug sensitivity in GC

The IC50 or semi-inhibitory concentration, serves as an important metric for estimating drug potency or treatment response. A lower IC50 concentration correlates with a better drug efficacy. In GC, individuals with high ANOS1 expression exhibited low IC50 concentrations for 5-fluorouracil, dasatinib, and docetaxel, suggesting enhanced treatment outcomes (Fig. 11A–C). Conversely, a high IC50 concentration for gefitinib indicated resistance to GC therapy (Fig. 11D).

## Discussion

Cancer remains one of the most devastating diseases worldwide, claiming millions of lives annually, especially gastric cancer (Mishra et al. 2023c). The KAL1 gene, located on chromosome Xp22.3 and spanning 67 kilobase pairs, encodes the ANOS1 protein, predominantly localized in the cytoplasm and possessing a molecular weight of 76 kDa (MacColl et al. 2002). ANOS1 functions as a secreted protein contributing to the ECM and is crucial for the migration of neuronal precursors. Furthermore, its involvement in tumorigenesis has been increasingly recognized in recent years. Analysis of data from the TCGA database revealed that ANOS1 expression levels was elevated in advanced GC tissues compared with normal tissues. The ROC analysis indicated a high diagnostic accuracy of ANOS1 in advanced GC, suggesting its significant role in disease progression and diagnostic utility. Furthermore, patients with GC exhibiting high ANOS1 expression levels had a poorer prognosis.

Immunohistochemical staining of advanced GC and adjacent tissue samples from 99 patients revealed an overexpression of ANOS1 in advanced GC. This finding was consistent with the data from the TCGA database. A significant association was observed between ANOS1 expression in advanced GC and various clinical pathological characteristics, including tumor infiltration, lymph node status, clinical TNM stage, and vascular invasion. Additionally, patients with advanced GC exhibiting ANOS1 overexpression had a significantly poorer OS compared with those with low ANOS1 expression. These results suggested that ANOS1 plays an important role in the progression of GC and may serve as a promising novel prognostic indicator for advanced GC.

Owing to the close association of ANOS1 expression with aggressive features and metastatic properties in advanced GC, we further examined the differential expression of ANOS1 between primary lesions and the corresponding lymph node metastases in patients with advanced GC. Our findings showed significantly higher ANOS1 expression in the lymph node metastatic tumors than primary tumors. These findings indicated that elevated ANOS1 expression may contribute to the invasive and metastatic behavior of advanced GC.

Moreover, we compared the ANOS1 differential expression across various histological types in patients with advanced GC. ANOS1 expression was significantly higher in the low-adhesion group compared with the high-adhesion group. These findings suggested a potential association between elevated ANOS1 expression and reduced tumor cell adhesion. Additionally, E-cadherin, an intercellular adhesion molecule, is closely linked to the impairment of tumor cell adhesion capability. Spearman’s correlation analysis showed that ANOS1 expression had a significant negative correlation with E-cadherin in advanced GC. These results suggested that the association between reduced tumor cell adhesion and ANOS1 overexpression in advanced GC may be related to the downregulation of E-cadherin expression.

To elucidate the biological mechanism of ANOS1 involvement in GC, a functional enrichment analysis was conducted. ANOS1 was predominantly enriched in pathways related to ECM receptor interaction, focal adhesion, hematological cell lineage, chemotherapy signaling, pathways in cancer, and the TGF-β signaling pathway in GC. A negative correlation was observed between ANOS1 expression and E-cadherin levels, prompting further investigation into the potential role of ANOS1 in the EMT process. E-cadherin, an intercellular adhesion molecule, is regulated by various transcription factors including SNAI1, TWIST1, and ZEB1/ZEB2 (Postigo et al. 2003; Barrallo-Gimeno and Nieto 2005; Yastrebova et al. 2021; Lamouille et al. 2014; Craene and Berx 2013; Yang et al. 2010). Vimentin and CDH2(N-cadherin) serve as crucial indicators of EMT, often exhibiting increased expression levels in conjunction with the suppression of E-cadherin expression (Yang et al. 2008; Zhang et al. 2013; Zheng et al. 2016; Liu et al. 2014; Mao et al. 2013). Analysis of the TCGA database revealed a significant positive correlation of ANOS1 mRNA levels with CDH2(N-cadherin), VIM, SNAL1, TWIST1, ZEB1, and ZEB2. These findings suggested a potential association between ANOS1 overexpression and EMT process in GC cells. Consequently, ANOS1 may contribute to diminished tumor cell adhesion through the downregulation of E-cadherin, thereby facilitating the EMT process.

The tumor immune microenvironment (TME) consists of tumor cells, tumor stem cells, infiltrating mesenchymal cells, immune cells, extracellular matrix, stromal tissue, microvasculature, and cytokines (Zheng et al. 2021). The TME plays a critical role in tumor differentiation, epigenetics, metastasis, and immune evasion. Our findings indicated a positive association between ANOS1 expression and infiltration of 20 immune cell types, as well as a negative correlation with two immune cell types in advanced GC. Notably, ANOS1 expression showed the strongest positive correlation with macrophages, suggesting the potential influence of ANOS1 expression on GC through the modulation of macrophage infiltration. The reports demonstrated that macrophages is closely associated with tumor proliferation, invasion, and metastasis, being a critical factor contributing to poor prognosis (Kitano et al. 2018; Zhou et al. 2019).The ESTIMATE algorithm identified a significant association between ANOS1 expression and stromal score, immune score, and ESTIMATE score in GC. The development of immune checkpoint blockers represents a transformative advancement in cancer treatment, with checkpoint inhibitor drugs demonstrating promising initial efficacy (Watanabe et al. 2020). Additionally, the expression of ANOS1 showed positive correlations with PDCD1LG2, HAVCR2, CD274, TIGIT, CTLA4, LAG3, and PDCD1. This findings suggested that ANOS1 played a role in regulating the activity of ICP genes through various signaling pathways. This indicated that ANOS1 may be a potential target for immunotherapy. Furthermore, our findings indicated that 5-fluorouracil, dasatinib, and docetaxel may improve treatment efficacy in patients for GC with elevated ANOS1 expression.

In conclusion, this study provides an integrative analysis of the role of ANOS1 in advanced GC. However, some limitations should be considered. We used bioinformatics methods to explore the correlation between ANOS1 and EMT-related genes. Further investigation is required to verify its relationship with EMT progress.

## Conclusion

Collectively, our research demonstrated the significant involvement of ANOS1 in the progression of GC, specifically in relation to tumor invasion and metastasis. Furthermore, our findings suggested a potential association between ANOS1 and EMT during GC progression. ANOS1 may be a novel prognostic indicator and a potential therapeutic target for the treatment of GC. However, there are some shortcomings in this study. The characteristics of ANOS1 were analyzed through bioinformatics and only conducted IHC to verify the overexpression of ANOS1 in advanced GC. However, there was no biological experiment to verify it. Therefore, in the future investigations, we adopt a systematic experimental approach to validate ANOS1's oncogenic mechanisms.

## Data Availability

The data are available from the corresponding author and the first author for reasonable requests.
